# Semi-Quantitative On-Site Microfluidic Assay to Detect 11-Nor-9-carboxy-delta 9-Tetrahydrocannabinol (THC-COOH) in Urine

**DOI:** 10.3390/s25237115

**Published:** 2025-11-21

**Authors:** YeJi Jung, Isaac Choi, Hyunjun Bae, Joonseok Seo, Sunchun Kim, Sangki Lee, Jeongmin Lee, Yohan Jeong, Juhyung Kim, Heesun Chung, Hyunho Kim, Seok Chung

**Affiliations:** 1 Department of Forensic Sciences, Sungkyunkwan University, Suwon 16419, Republic of Korea; jungyeji03@gmail.com (Y.J.); skleedoc@naver.com (S.L.); 2 KU-KIST Graduate School of Converging Science and Technology, Korea University, Seoul 02841, Republic of Korea; isack7754@gmail.com; 3 Absology Ltd., Anyang 14057, Republic of Koreajsseo@absology.co.kr (J.S.); jmlee@absology.co.kr (J.L.); yhjeong@absology.co.kr (Y.J.); jhkim@absology.co.kr (J.K.); 4 National Forensic Service, Wonju 26460, Republic of Korea; pharm88@nate.com; 5 School of Mechanical Engineering, Korea University, Seoul 02841, Republic of Korea; 6 Center for Brain Technology, Brain Science Institute, Korea Institute of Science and Technology (KIST), Seoul 02792, Republic of Korea

**Keywords:** THC-COOH, biosensor, point-of-care testing (POCT), semi-quantitative analysis, fluorescence-based LFA, microfluidic cartridge, lab-on-a-chip (LOC), matrix effect, forensic toxicology, portable analytical device

## Abstract

**Highlights:**

- Developed a portable microfluidic device for rapid (4 min) on-site detection of THC-COOH at a low cutoff of 20 ng/mL.
- Device validation using 100 authentic urine samples showed 100% sensitivity/specificity and high correlation (R^2^ = 0.9471) with LC-MS/MS.

**What are the main findings?**

- A test-to-reference (T/R) fluorescence ratio was successfully employed to mitigate urine sample matrix effects and ensure objective, normalized semi-quantitative results.
- The antibodies used in the cartridge were found to recognize both free THC-COOH and its primary glucuronide-conjugated metabolite, eliminating the need for enzymatic hydrolysis pre-treatment.

**What are the implications of the main findings?**

- The device provides objective, numerical readouts and digital data management, which overcomes the critical limitations of subjective visual interpretation and poor data integrity found in conventional rapid kits.
- The system’s design enables future integration of creatinine measurement for urine validity testing, suggesting a pathway to a comprehensive on-site device that fully meets forensic toxicology standards.

**Abstract:**

The rapid detection of 11-nor-9-carboxy-delta-9-tetrahydrocannabinol (THC-COOH), a primary cannabis metabolite, is critical for forensic and workplace drug testing. However, conventional immunoassays often lack sensitivity and objectivity. We developed a portable lateral flow immunoassay device with a microfluidic cartridge and fluorescent reader for the semi-quantitative detection of THC-COOH in urine. A test-to-reference fluorescence ratio was employed to mitigate matrix effects and ensure objective results. The device was validated for accuracy, repeatability, and stability using spiked urine samples and compared against validated LC-MS/MS results on 100 authentic urine samples (50 positive and 50 negative). At a cutoff of 20 ng/mL, the device achieved 100% sensitivity and specificity, with repeatability and reproducibility CVs of below 15%. The cutoff index (COI) strongly correlated with LC-MS/MS results (R^2^ = 0.9471). Crucially, this high correlation with hydrolyzed LC-MS/MS data demonstrates that the antibody recognizes both free and glucuronide-conjugated metabolites, validating its reliability without enzymatic pre-treatment. This microfluidic device enables rapid, sensitive on-site THC-COOH detection, featuring automated data management via Wi-Fi connectivity, enhancing its forensic applicability.

## 1. Introduction

Cannabis is one of the most widely used drugs worldwide and its use is continuously increasing. Over the past decade, the global number of cannabis users has increased by approximately 34%. As of 2023, it is estimated that 244 million people used cannabis, corresponding to 4.6% of the global population aged 15–64 [1]. Recently, there has been a shift in the perception of the legal status and medical value of cannabis. In 2020, the United Nations rescheduled cannabis from ‘Schedule IV’ to ‘Schedule I’ narcotics, acknowledging its potential for medical or therapeutic use [2,3,4]. Consequently, as of 2021, 64 countries have permitted the use of cannabinoid pharmaceuticals and/or cannabis-based products for medical purposes. Of these, 34 countries allow the use of cannabis-based products for the treatment of various medical conditions [5].

Cannabis consumption has diversified from traditional smoking to vaping and edible forms such as brownies, chocolate, jellies, and beverages. Particularly, edible cannabis products pose a higher risk of unintentional ingestion and overconsumption [6,7]. These developments have made cannabis use easier to conceal. Research demonstrates a correlation between cannabis use and reduced driving performance, which is further exacerbated when combined with alcohol, emphasizing the need for effective cannabis screening before driving [8,9,10,11]. In the United States, the rate of positive cannabis tests among general workers has shown a continuous upward trend in recent years [12]. Cannabis abuse testing using urine samples typically consists of two stages: an initial drug screening and a confirmatory test. Initial drug screening primarily utilizes immunoassays to rapidly assess the possibility of drug use. If the initial drug screen is positive, a confirmatory test using analytical techniques, such as GC-MS/MS or LC-MS/MS, is conducted. Confirmatory tests have the advantage of accurately identifying and quantitatively measuring specific drugs but require complex sample preparation in specialized laboratories, leading to longer turnaround times [13].

Immunoassays include rapid diagnostic kits for simple on-site use and laboratory equipment capable of testing a large number of samples [13]. Rapid diagnostic kits (rapid kits), which typically rely on colorimetric changes induced by nanoparticle conjugates, are prone to significant inaccuracies. As observed during the COVID-19 pandemic, the interpretation of results from such kits can vary depending on the individual reading them, the characteristics of the specimen, and the timing of the analysis. Consequently, careful attention is required when interpreting their results [14]. Furthermore, rapid kits require manual recording of results or photographing and uploading data, which can be cumbersome and may lead to data loss or errors. Large-scale immunoassay equipment processes a large number of samples but involves constraints such as high costs, maintenance difficulties, and the need for specialized personnel [13]. Therefore, there is a critical and unmet need for a ‘smart’ sensor system that bridges the gap between inaccurate disposable kits and complex laboratory instruments—one that is portable, quantitative, cost-effective, and provides objective, digitally managed results.

Herein, we develop and validate a portable, fluorescence-based microfluidic immunoassay system that overcomes the limitations of both subjective rapid kits and centralized laboratory equipment. The objective was to create a rapid (4 min), sensitive, and robust point-of-care testing (POCT) platform suitable for forensic applications. Using 11-nor-9-carboxy-delta-9-tetrahydrocannabinol (THC-COOH) [15], the primary metabolite of tetrahydrocannabinol (THC), which is the main psychoactive component of Cannabis sativa plant [16], as a model analyte, our device demonstrates a low cutoff of 20 ng/mL and achieves 100% sensitivity and 100% specificity when validated against 100 authentic forensic urine samples. Furthermore, the system provides objective, semi-quantitative results (R^2^ = 0.9471 vs. LC-MS/MS) and features automated data management, offering a complete and reliable solution for on-site drug screening.

## 2. Materials and Methods

### 2.1. Microfluidic Cartridge Fabrication

**Figure 1:**
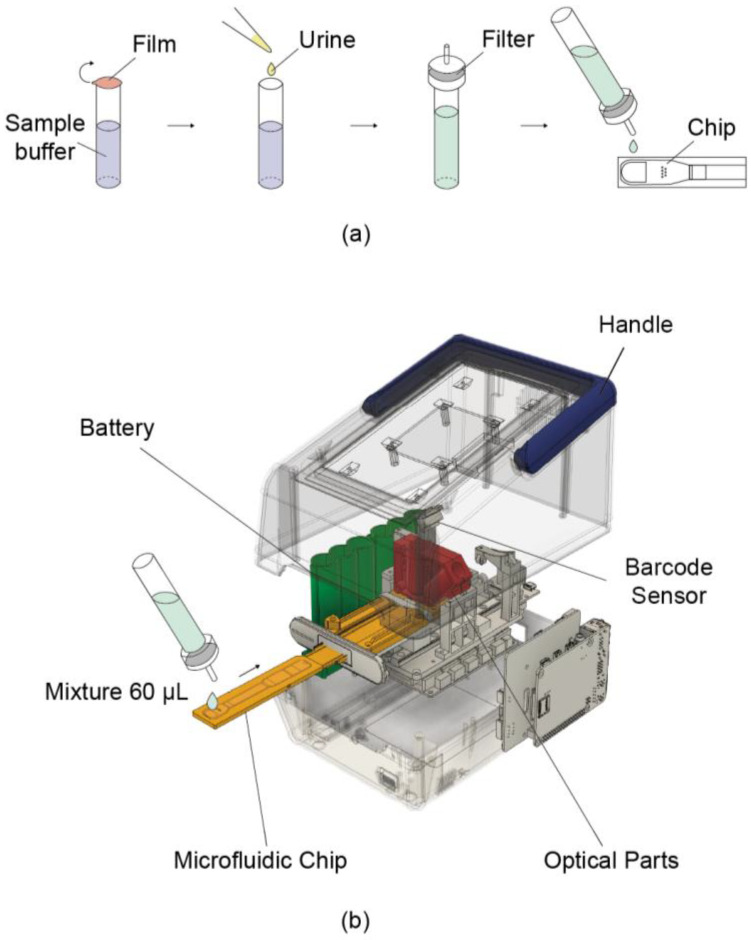
Schematic illustration of the portable reader and its usage procedure. (**a**) Sample pretreatment process. Urine is mixed with the buffer contained in the tube at a 1:10 ratio. A dropper cap with a built-in filter is then attached, and the mixture is loaded onto the chip. (**b**) Schematic of the device operation. A 60 μL mixture is loaded onto the chip, which is then reacted inside the device for 4 min. Fluorescence is measured via the built-in optical parts, and the concentration is determined accordingly.

PMMA (polymethyl methacrylate) was used as cartridge material. Before assembly, injection molded parts were treated with plasma (500 SCCM of O_2_ flow for 320 s at 0.7 kW RF and 0.14 Torr). To stabilize fluid flow, a nano-interstice structure was introduced [17,18]. Prior to loading, the sample was mixed with a sample buffer at a 1:10 ratio and then applied to the inlet of the cartridge (Figure 1a). After loading, the cartridge was allowed to operate in the reader (Figure 1b).

#### 2.1.1. Streptavidin Conjugation

**Figure 2:**
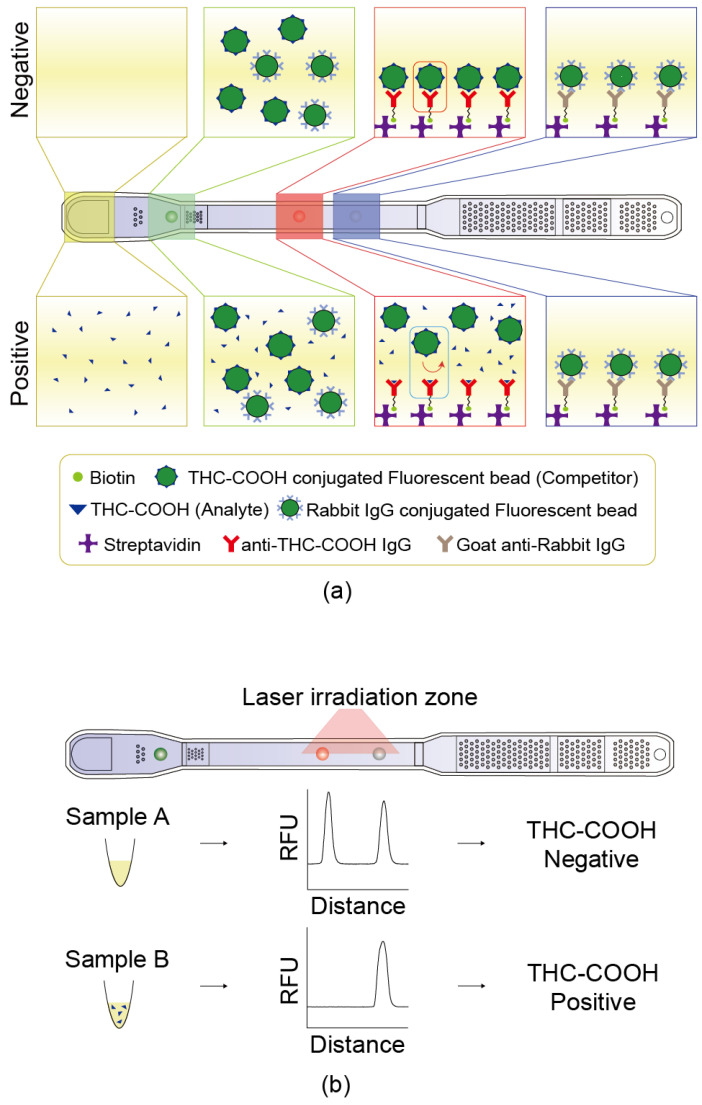
Principle of the competitive microfluidic immunoassay. (**a**) Schematic illustration of competitive binding reaction occurring inside the chip, depicting the mechanism for both THC-COOH-negative and -positive urine samples. (**b**) Representative fluorescence intensity graphs obtained after the fluorescent beads have reacted on the chip, showing differences in signal depending on the presence or absence of THC-COOH.

Streptavidin was used as a linker to immobilize anti-THC-COOH IgG (test line, Figure 2a, red box) and goat anti-rabbit IgG (reference line, Figure 2a, blue box).

Streptavidin was conjugated onto the PMMA surface via the 1-ethyl-3-(3-dimethylaminopropyl)carbodiimide (EDC)/N-hydroxysuccinimide (NHS) reaction. To facilitate covalent immobilization of streptavidin onto the surface, a mixture of streptavidin (100 µg/mL), 3 mM EDC, and 3 mM NHS in 50 mM 2-(N-morpholino) ethanesulfonic acid (MES) buffer (pH 6.2) was incubated for 1 h at room temperature to enable amide bond formation between surface carboxyl groups and streptavidin amines. After activation, 2 µL of the streptavidin solution was dotted onto the test and reference dots using a pipette. To prevent evaporation, the PMMA cartridge surface was incubated in a humidified chamber for 1 h. Unbound streptavidin was removed using washing buffer (Cat. #AWB1, Absology), and the surface was dried at 15–25 °C for 1 h.

#### 2.1.2. Capture Antibody Immobilization

Antibody biotinylation was performed to allow binding to the streptavidin previously immobilized on the surface of the lower plate of the cartridge. Biotin (Cat. #21338, Thermo Fisher Scientific, Waltham, MA, USA) was added to phosphate-buffered saline (PBS) containing the antibody at a 20-fold molar ratio and allowed to react for 1 h. Excess biotin was removed using a dialysis cassette (cat. #87734, Thermo Fisher) against 5 L of PBS, which was replaced more than seven times at intervals of at least 1 h over a 16 h period. The biotinylated antibody was dotted onto the streptavidin-coated surface of the lower plate. The test IgG solution was prepared by diluting the biotinylated anti-THC-COOH IgG complex to 100 µg/mL in distilled water and mixing it with dotting buffer (Cat. #ADTB, Absology, Anyang, Republic of Korea). A 1.2 µL aliquot of this solution was pipetted onto the plate’s surface (Figure 2a, red box). For the reference, a solution of biotinylated anti-rabbit IgG was prepared at 50 µg/mL in distilled water, mixed with dotting buffer (Cat. #ADTB, Absology), and 1.2 µL was dotted onto the surface (Figure 2a, blue box). The dotted chip was dried at 37 °C for 1 h. Unbound components were removed using a washing buffer (Cat. #AWB2, Absology), and the dotted lower plate was dried again at 5–25 °C for 1 h.

#### 2.1.3. Detection Antibody–Fluorescent Bead Conjugation

Two types of fluorescent beads were dotted onto the surface of the lower plate: a test bead conjugated with THC-COOH/Bovine Serum Albumin (BSA) and a reference bead conjugated with rabbit IgG. The test bead competed with THC-COOH present in the sample and bound to the anti-THC-COOH IgG located at the test dot. The reference bead was used to compensate for variations in fluorescence intensity that may arise from differences among human urine samples. It bound to goat anti-rabbit IgG immobilized on the reference dot (Figure 2a). Test beads were synthesized via an EDC/NHS reaction between THC-COOH/BSA and fluorescent beads. A 0.2% solution of 0.5 µm fluorescent beads (Cat. #T7281, Thermo Fisher Scientific) was prepared in 50 mM MES buffer (pH 6.2) containing 3 mM EDC (Cat. #22980, Thermo Fisher Scientific) and 3 mM NHS (Cat. #56485, Sigma-Aldrich, St. Louis, MO, USA). The bead solution was activated by mixing it in a roller mixer for 30 min, followed by the addition of THC-COOH/BSA and a reaction time of 2 h. The solution was then blocked with 1% BSA and centrifuged at 21,055× *g* for 15 min. The supernatant was discarded, and the pellet was resuspended in storage buffer (Cat. #ASTB, Absology). Reference beads were synthesized by conjugating rabbit IgG to fluorescent beads using EDC/NHS chemistry. A 0.2% solution of 0.5 µm fluorescent beads (Cat. #T7281, Thermo Fisher Scientific) was prepared in a 50 mM MES buffer (pH 6.2) containing 3 mM EDC and 3 mM NHS. The bead solution was mixed on a roller mixer for 30 min for activation, followed by a 2 h reaction with rabbit IgG. The beads were then blocked with 1% BSA, centrifuged at 21,055× *g* for 15 min, and resuspended in storage buffer (Cat. #ASTB, Absology). The prepared fluorescent beads were dotted on the surface of the lower plate. For the dotting solution, a 0.3% solution of the fluorescent bead–antibody (or antigen) complex was prepared in distilled water, mixed with fluorescent bead conjugation buffer (Cat. #ACJB, Absology) (Figure 2a, green box).

#### 2.1.4. Cartridge Assembly and Channel Formation

The lower plate, immobilized with the antibodies and fluorescent beads, was bonded to an upper plate to form a cartridge. Acetone–chloroform mixture was injected into the holes of the upper plate, followed by pressing with a mechanical press with 0.5 MPa pressure to complete assembly.

### 2.2. Detection and Analysis

#### 2.2.1. Preparation for Detection

A urine sample was mixed with sample buffer at a 1:10 ratio, and 60 µL of the diluted solution was loaded onto the prepared cartridge. The cartridge was inserted into a reader (dimensions of 127 mm × 206 mm × 150 mm and a weight of 1.5 kg). The loaded urine sample flowed through the channel of the cartridge and reacted with the fluorescent beads at the test and reference dots for 4 min.

#### 2.2.2. Fluorescence Measurement

Laser light excited fluorescent labels, and the emitted fluorescence was measured by photodiode (Figure 2b). The measured intensity at the test dot correlated to the concentration of THC-COOH in the sample, whereas that at the reference dot reflected sample matrix effects, including interference due to urine composition. T/R ratio, defined as the test intensity divided by the reference intensity, was used as the normalized signal to determine the THC-COOH concentration in the urine sample.

#### 2.2.3. Definition of THC-COOH Concentration

The cutoff T/R ratio was measured using a standard material and then divided by the sample T/R ratio. A value greater than 1 indicates a concentration above the cutoff. Concentrations of THC-COOH and THC-COOH-conjugated fluorescent beads were estimated through a competitive reaction in urine. Consequently, a reverse proportionality graph was obtained, where higher concentrations corresponded to lower sample test/ref ratios. A linear concentration graph can be obtained by taking the reciprocal of this value, referring to THC-COOH Cutoff Index (hereinafter abbreviated as COI).

THC−COOH COI=1sampleTRratiocut offTRratio

### 2.3. Reliability Evaluation

Performance evaluation was conducted under laboratory-developed test (LDT) protocol in a license holder, Expertox Lab (Deer Park, TX, USA), to assess standard testing, accuracy, operator-to-operator variability, lot-to-lot variability, instrument-to-instrument variability, repeatability, limit of detection (LOD), and limit of blank (LOB). For standard testing, samples were prepared by spiking blank urine with a THC-COOH reference standard (Cerilliant, T-101) at concentrations of 25, 50, 75, 100, and 1000 ng/mL. The cutoff ratio used in the COI (cutoff index) was at a concentration of 20 ng/mL. The reproducibility (operator-to-operator, lot-to-lot, instrument-to-instrument) and repeatability of the device were assessed; low-, medium-, and high-concentration samples were prepared by dissolving the THC-COOH standard (Cat. # T-019, Cerilliant, Round Rock, TX, USA) in negative buffer composed of 1X PBS (pH 7.4) and 0.05% sodium azide. The concentrations of the prepared samples were 50 ng/mL (low), 100 ng/mL (medium), and 1000 ng/mL (low, medium, and high, respectively), and their cutoff value was 20 ng/mL. Three lots (584902, 584903, and 584904) were tested, with ten replicates for each lot at each of the three concentrations (low, medium, and high), for a total of 30 tests per lot. An operator-to-operator test was performed using lot 584902 by three operators, conducting four tests each per concentration (low, medium, and high) per day for five consecutive days: 60 tests per operator in total. Three lots (584902, 584903, and 584904) were used for the lot-to-lot tests. For each lot, four tests per concentration were conducted per day for five days, resulting in 60 tests per lot. The instrument-to-instrument test was conducted using lot 584902 across three different instruments. Each instrument was used to perform four tests per concentration per day over five days, for a total of 60 tests per instrument. The LOB samples consisted of a negative buffer [1X (10 mM) PBS (pH 7.4), 0.05% sodium azide], whereas the LOD samples were prepared by spiking the same negative buffer with 50 ng/mL of the THC-COOH reference standard (Cat# T-109, Cerilliant).

### 2.4. Urine Sample Collection and Analysis

One hundred urine samples (fifty positive and fifty negative for cannabis) were obtained from the National Forensic Service (NFS) of South Korea. The cutoff concentration was set at 20 ng/mL. This target was chosen to achieve higher sensitivity than the conventional 50 ng/mL screening cutoff concentration (specified by guidelines such as SAMHSA [19]). This 20 ng/mL cutoff is an established high-sensitivity screening threshold used in clinical and forensic monitoring [20,21] and is consistent with the trend toward stricter standards (e.g., 25 ng/mL used by the NFS [22]). This cutoff closely approaches the 15 ng/mL confirmation cutoff concentration [19].

### 2.5. LC-MS/MS Quantitative Analysis

#### 2.5.1. Chemicals and Reagents

THC-COOH (1.0 mg/mL in methanol) and the internal standard THC-COOH-d_3_ (100 μg/mL in methanol) were purchased from Cerilliant Corporation (Round Rock, TX, USA). LC-MS-grade formic acid was obtained from Supelco (St. Louis, MO, USA), HPLC-grade water was obtained from Fisher Chemical (Waltham, MA, USA), and HPLC-grade acetonitrile was obtained from Honeywell (Morris Plains, NJ, USA). Monobasic and dibasic sodium phosphates were purchased from Samchun Chemicals (Pyeongtaek, Gyeonggi-do, Korea). β-glucuronidase (derived from *E. coli* K12) was acquired from Roche Diagnostics (F. Hoffmann-La Roche Ltd., Basel, Switzerland). Hexane, ethyl acetate, and methanol were purchased from Sigma-Aldrich (St. Louis, MO, USA), Duksan Pure Chemicals (Ansan-si, Gyeonggi-do, Korea), and J.T. Baker (Avantor, Radnor, PA, USA), respectively.

#### 2.5.2. Method Development

A SCIEX 3200 QTRAP MS system (Framingham, MA, USA) coupled with an Agilent 1260 Infinity Binary HPLC system (Santa Clara, CA, USA) was optimized to simultaneously detect THC-COOH and the internal standard THC-COOH-d_3_. Multiple Reaction Monitoring (MRM) transitions for quantitative analysis were 345.21/299.20 (*m*/*z*) for THC-COOH and 348.21/330.20 (*m*/*z*) for THC-COOH-d_3_, with a dwell time of 200 ms and a source temperature of 600 °C. Liquid chromatographic separation was performed using a Poroshell 120 EC-C18 column (2.1 × 100 mm, 2.7 μm, Agilent Technologies, Santa Clara, CA, USA) at 40 °C. The mobile phase used for LC-MS/MS analysis was a gradient elution. Mobile phase A was water with 0.1% formic acid and mobile phase B was acetonitrile with 0.1% formic acid. The gradient started at 70% A and 30% B, held for 2 min, linearly shifted to 20% A and 80% B over 9 min, maintained for 10 min, and rapidly adjusted to 80% A and 20% B for 10.5 min. From 11 min to 16 min, the system was re-equilibrated to 70% A and 30% B. The total run time was 16 min, with a flow rate of 450 μL/min. MRM data acquired by LC-MS/MS were processed using the Analyst software (version 1.6.3, SCIEX, Framingham, MA, USA).

#### 2.5.3. Urine Sample Preparation and THC-COOH Extraction

In a 2 mL tube, 300 μL of urine sample was mixed with 10 μL of the internal standard (THC-COOH-d_3_, 300 ng/mL) and 100 μL of 0.2 M phosphate buffer (pH 6.0). To hydrolyze glucuronide conjugates and isolate free THC-COOH, 10 μL of β-glucuronidase was added, and the mixture was incubated at 48 °C for 60 min. After hydrolysis, 1.2 mL of the extraction solvent (hexane/ethyl acetate = 4:1) was added, and the mixture was vortexed for 10 min using a multi-tube vortex mixer. After centrifugation at 13,000 rpm for 10 min, the supernatant was separated and evaporated under nitrogen. The dried residue was reconstituted in 50 μL of methanol, and after centrifugation, 10 μL of the supernatant was injected into the LC-MS/MS system to quantify THC-COOH. For samples without enzyme treatment, the phosphate buffer and β-glucuronidase steps were omitted, and the extraction solvent (hexane/ethyl acetate = 4:1) was directly added, followed by the same extraction procedure to isolate free THC-COOH. The purpose of analyzing samples with and without β-glucuronidase treatment was to determine whether the antibodies bind solely to free THC-COOH or to glucuronide-bound THC-COOH in the urine sample.

#### 2.5.4. Method Validation and Sample Analysis

LC-MS/MS method validation included tests for selectivity, accuracy, precision, recovery, matrix effect, and carryover of THC-COOH in urine samples. Calibration curve samples (5, 20, 60, 120, and 200 ng/mL) and quality control (QC) samples (15, 50, and 180 ng/mL) were prepared and analyzed separately using THC-COOH-free human urine. Sample with concentrations exceeding the upper limit of the calibration curve (200 ng/mL) were diluted with THC-COOH-free human urine to bring the concentration within the calibration range. The final concentration was calculated by applying the dilution factor. Subsequently, cannabis-positive urine samples provided by the NFS were analyzed, with and without enzyme treatment during sample preparation, to determine THC-COOH concentrations.

### 2.6. Correlation of Absol Device Analysis with LC-MS/MS Results

A correlation analysis was performed between the COI values measured by the developed method of cannabis-positive samples (Section 2.3) and THC-COOH concentrations measured by LC-MS/MS (Section 2.4).

## 3. Results

### 3.1. Reliability and Precision Evaluation Under Laboratory-Developed Test Protocol

**Figure 3:**
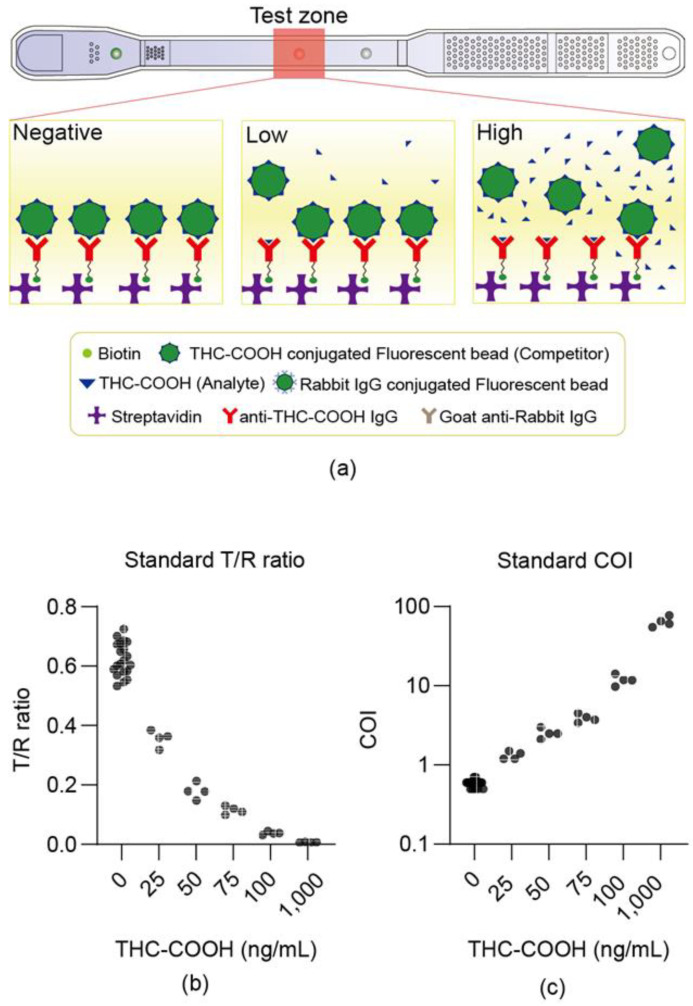
Device dose–response to THC-COOH standards in urine. (**a**) Shows the degree of fluorescence bead binding according to the THC-COOH concentration. (**b**,**c**) Show the measurement results of the LDT standard material, where the test was conducted by mixing the standard material with the negative urine samples. (**b**) Illustrates the correlation between the test/ref ratio and concentration of the THC-COOH standard material. (**c**) Shows the correlation between the COI and the concentration of the THC-COOH standard material, demonstrating the wide dynamic range (0–1000 ng/mL) of the microfluidic device. This curve is distinct from the calibration curve (5–200 ng/mL) used to validate the LC-MS/MS reference method (Section 2.5.4).

This device estimates concentration based on a competitive reaction mechanism (Figure 3a). Reliability was first checked at Expertox Lab (TX 77536, USA), which holds a laboratory-developed test (LDT) license. We measured samples prepared by spiking the THC-COOH standard into negative urine, and the T/R ratio, which is proportional to the number of fluorescent beads bound to the test dot, was found to be inversely proportional to the concentration (Figure 3b). This inverse relationship (Figure 3b) is characteristic of competitive immunoassays, which typically follow an inverse sigmoidal dose–response curve [23]. By optimizing the assay parameters for the operational range of interest, we minimize saturation effects, enabling the use of a simple reciprocal plot (COI) for effective linearization. When the sample concentration measured exceeded the cutoff level of 20 ng/mL, the COI value was above 1. The measured COI values made a good correlation with the concentration across a range of values.

**Figure 4:**
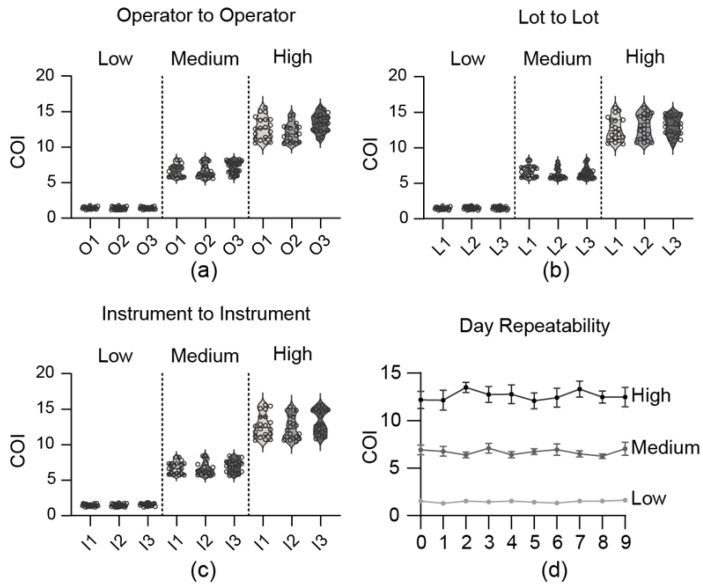
Reliability and precision evaluation (LDT results). Results obtained by mixing THC-COOH standard material with 1X PBS buffer. (**a**) Operator-to-operator reproducibility. (**b**) Lot-to-lot reproducibility. (**c**) Instrument-to-instrument reproducibility. (**d**) Repeatability over 9 days.

The accuracy, operator-to-operator, lot-to-lot, instrument-to-instrument, and repeatability tests were conducted at low (50 ng/mL), medium (100 ng/mL), and high (1000 ng/mL) concentrations to confirm the reliability. For operator-to-operator variability (Figure 4a), the within-operator CV was less than 15%, and the between-operator CV was within 14%. The lot-to-lot variability (Figure 4b) showed within-lot CVs under 15%, and between-lot CVs within 13%. Instrument-to-instrument variability (Figure 4c) demonstrated within-instrument CVs of less than 14% and also between-instrument CVs of less than 14%. Furthermore, a one-way ANOVA confirmed this robustness. No statistically significant differences (*p* > 0.05) were found among the group means for the lot-to-lot or instrument-to-instrument comparisons. While a statistical difference was noted in the operator-to-operator (High) test (*p* = 0.001), the between-operator CV for this group remained low at 14%, confirming that all reproducibility metrics were well within the standard acceptance criteria (CV < 15%). Repeatability, evaluated by conducting four tests per day, demonstrated CV values of less than 20% across the low, medium, and high concentrations. Over a 10-day period, the overall CV values were 11.7%, 12.9%, and 12.8% for low, medium, and high concentrations, respectively, indicating that stability was maintained (Figure 4d). The LOB was measured 60 times, yielding an average of 0.44 with a CV of 14.6%, whereas the LOD, measured at 50 ng/mL, showed an average of 1.00 with a CV of 14.9%.

In summary, the comprehensive LDT evaluation confirmed the high reliability, precision, and stability of the developed microfluidic device for THC-COOH detection under controlled laboratory conditions.

### 3.2. Field Applicability Test Results

**Figure 5:**
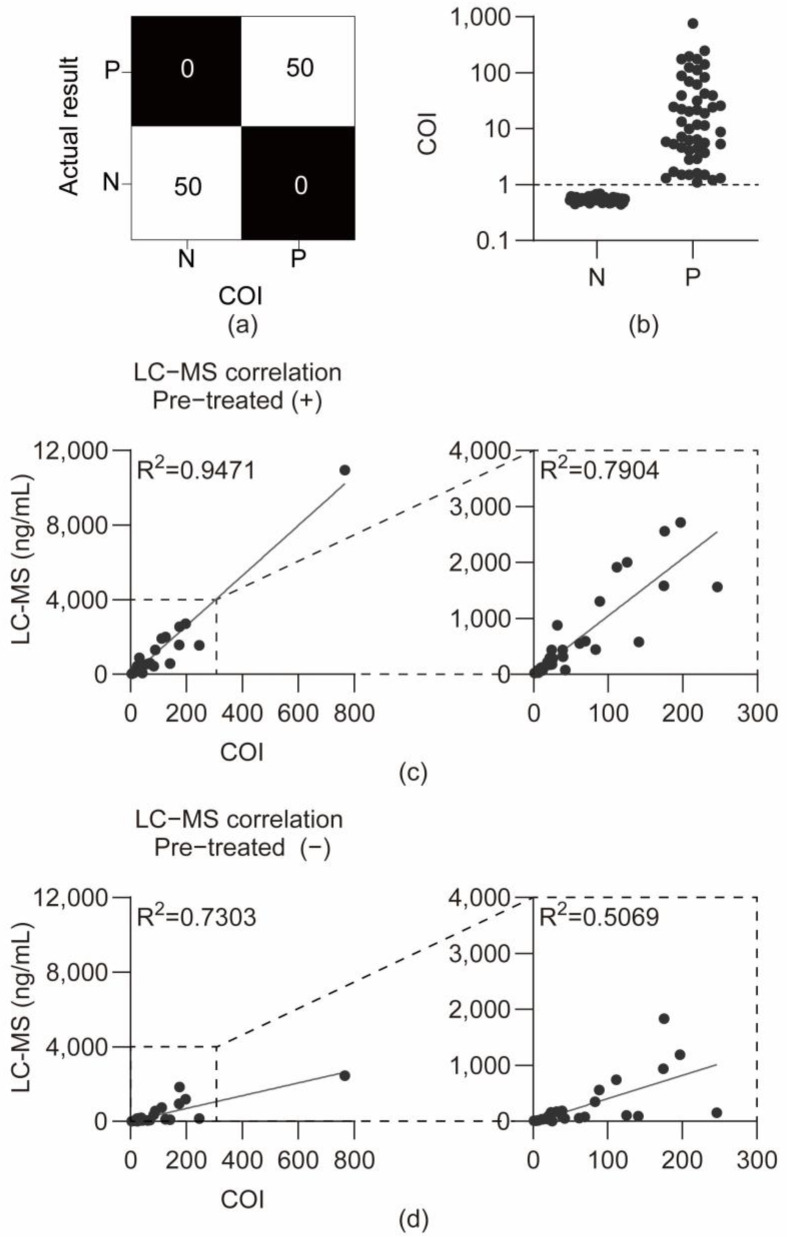
Analysis of authentic urine samples. (**a**) Confusion matrix comparing COI-based test results with LC-MS/MS reference results. (**b**) Graph of COI values for negative and positive samples from the National Forensic Service. (**c**) Correlation between LC-MS/MS concentrations and COI values for enzyme-treated positive samples and correlation graph excluding the highest concentration sample among enzyme-treated positive samples. (**d**) Correlation between LC-MS/MS concentrations and COI values for non-enzyme-treated positive samples and correlation graph excluding the highest concentration sample among non-enzyme-treated positive samples.

The reliability of the developed device was first confirmed under LDT protocols, and then its applicability was checked under the regulation. One hundred urine samples were tested, including 50 THC-negative and 50 THC-positive samples, obtained from the National Forensic Service. The cutoff level was set at 20 ng/mL. This high-sensitivity cutoff level is lower than the conventional 50 ng/mL screening cutoff level [19] and similar to other strict forensic screening standards (e.g., 25 ng/mL [22] and 20 ng/mL [20,21]), and was used to evaluate the device’s applicability in authentic samples. Zero false negatives and zero false positives were confirmed, demonstrating 100% specificity and 100% sensitivity (Figure 5a,b).

These results demonstrate the excellent diagnostic accuracy (100% sensitivity and 100% specificity) of the device when applied to authentic forensic urine samples, validating its potential for reliable field screening against the established cutoff.

### 3.3. Correlation Between LC-MS/MS Concentrations and COI Values

Quantification of THC-COOH in human urine was validated using an LC–MS/MS method with a standard calibration curve (5–200 ng/mL). Method validation was evaluated for selectivity, accuracy, precision, recovery, matrix effects, and carryover in two sample preparation approaches (with and without enzymatic hydrolysis). All validation parameters satisfied the predefined acceptance criteria. Detailed results are presented in Tables S1–S3. The concentration of THC-COOH was measured with and without enzyme treatment using a validated LC-MS/MS method on 50 cannabis-positive authentic urine samples, to prove that the THC-COOH antibody used in the cartridge bound to free THC-COOH, as well as to acid-linked THC-COOH glucuronide conjugates. In enzyme-treated urine samples, THC-COOH concentrations ranged from a minimum of 7.557 ng/mL to a maximum of 10,950 ng/mL, whereas in non-enzyme-treated urine samples, THC-COOH concentrations ranged from a minimum of 5.257 ng/mL to a maximum of 2458 ng/mL. One sample exhibited a notably high concentration of THC-COOH (10,950 ng/mL) after enzyme treatment.

The sample-specific COI values and LC-MS/MS quantification data are presented in Table S4. As shown in Figure 5b, the COI values provided a clear diagnostic separation between the 50 negative (N) and 50 positive (P) authentic samples, confirming the 100% diagnostic sensitivity and specificity at the cutoff index of 1.0. The COI value, defined as the reciprocal of the T/R ratio, exhibited a significant positive correlation with the concentrations measured by LC-MS/MS (R^2^ = 0.9471, *p* < 0.0001) (Figure 5c). Additionally, a correlation with LC-MS/MS measurements conducted without enzymatic treatment was confirmed (R^2^ = 0.7303, *p* < 0.0001) (Figure 5d). After excluding a sample with a notably high concentration, the correlation analysis revealed that the correlation coefficient for enzyme-treated samples was (R^2^ = 0.7904, *p* < 0.0001), whereas that for non-enzyme-treated samples was (R^2^ = 0.5069, *p* < 0.0001). The strong correlation between device COI values and LC-MS/MS measurements with enzymatic hydrolysis (R^2^ = 0.9471) validates the semi-quantitative performance of the assay. This high correlation demonstrates that the antibody recognizes both free and glucuronide-conjugated THC-COOH forms. Consequently, the device can provide reliable total THC-COOH quantification without requiring enzymatic pre-treatment—a significant practical advantage for field deployment. The lower correlation with non-enzyme-treated LC-MS/MS data (R^2^ = 0.7303) is consistent with this interpretation, as it reflects the expected difference between measuring total versus free THC-COOH concentration. This distinction is further explored in the Section 4.

### 3.4. Reader Configuration

**Figure 6:**
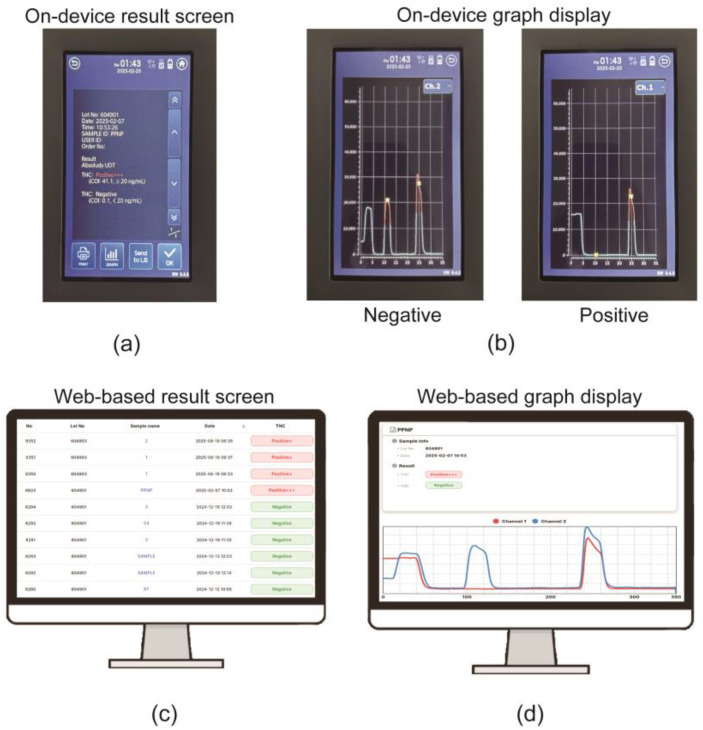
Reader interface and data management system. (**a**) On-device screen displaying a final semi-quantitative result. (**b**) On-device graph display showing examples of ‘Negative’ (high test peak) and ‘Positive’ (low test peak) results. (**c**) Web-based data management screen listing consolidated results. (**d**) Web-based screen showing detailed graph data, demonstrating the Wi-Fi data transmission. (+) [20 ng/mL], (++) [50 ng/mL], (+++) [100 ng/mL].

Upon completion of the concentration measurement, the reader display indicated the THC-COOH concentration levels as Negative or Positive {(+)[20 ng/mL], (++)[50 ng/mL], (+++)[100 ng/mL]}, along with a fluorescence graph corresponding to the position (Figure 6a,b). The reader was designed to connect to Wi-Fi to upload the fluorescence measurement graph and THC-COOH concentration levels (Negative, Positive {(+), (++), (+++)}), enabling consolidated result management (Figure 6c,d). This integrated readout and connectivity enhance the practicality and reliability of the device for on-site applications, where objective interpretation and robust data handling are crucial.

## 4. Discussion

The key innovation of this study lies in the successful integration of a sensitive fluorescence-based microfluidic assay with a robust internal reference system, thereby establishing a portable sensor platform that directly bridges the gap between unreliable disposable kits and complex laboratory equipment. Conventional dye-based rapid kits suffer from fundamental limitations due to subjective visual readings and low sensitivity (typically 50 ng/mL) [14]. This system addresses these critical shortcomings in two ways. Firstly, it provides the quantitative signal required to lower the detection limit to a forensically meaningful level of 20 ng/mL using fluorescence-based LFA. Second, more crucially for a ‘smart sensor’ operating in variable biological matrices, it effectively mitigates the high variability of urine matrix effects by integrating a reference bead channel to generate a normalized test-to-reference (T/R) ratio (COI). This on-chip normalization is the key to transforming from a simple ‘yes/no’ test to an objective semi-quantitative screening tool, whose reliability is demonstrated by high correlation with LC-MS/MS (R^2^ = 0.9471) and 100% diagnostic accuracy across 100 authentic forensic samples. Importantly, the COI is the intended semi-quantitative endpoint of the device; converting this normalized ratio back to an absolute concentration (ng/mL) using a mathematical standard curve (such as Figure 3c) is not the device’s intended function and would be scientifically inappropriate. Such conversion would reintroduce the sample-specific matrix effects that the T/R normalization ratio was specifically designed to mitigate. Therefore, the device operates optimally within its designed semi-quantitative diagnostic framework (Negative, +, ++, +++).

In urine samples, the cannabis metabolite THC-COOH is present in both free and glucuronide-conjugated forms. THC-COOH is generated through oxidative metabolism of THC and is subsequently conjugated with glucuronic acid to enhance solubility, with acid-linked THC-COOH glucuronide being the primary urinary metabolite [15,24]. To evaluate whether immunoassays can be performed without hydrolysis, authentic cannabis-positive urine samples were analyzed using validated LC-MS/MS methods, both with and without β-glucuronidase treatment. The measured THC-COOH concentrations were compared with the COI values obtained from the developed device. When glucuronide-bound THC-COOH was hydrolyzed to its free form, the correlation between LC-MS/MS concentrations and COI values was high (R^2^ = 0.9471). In contrast, when only free THC-COOH was measured without hydrolysis, the correlation decreased significantly (R^2^ = 0.7303). To minimize the effect of extreme values, a sample with an exceptionally high THC-COOH concentration (10,950 ng/mL) was excluded from the analysis. This exclusion yielded the correlation coefficients R^2^ = 0.7904 for enzyme-treated samples and R^2^ = 0.5069 for non-enzyme-treated samples, indicating sensitivity to outliers but retention of statistical significance within the typical concentration range. Exceptionally high THC-COOH levels may occur following consumption of high-potency cannabis, edible cannabis products, or vaporized THC oil [25].

These findings suggest that the antibody used in the cartridge recognizes both free and glucuronide-bound THC-COOH, allowing the developed device to detect glucuronide-bound forms without hydrolysis. The device reports THC-COOH concentrations semi-quantitatively using a number of (+) symbols, enabling a more refined estimation of both the timing and extent of cannabis use. This semi-quantitative capability provides valuable information beyond binary positive/negative results, supporting decision-making in law enforcement and workplace drug testing. Compared with quantitative LC-MS/MS, the device offers a cost-effective, rapid, and field-applicable alternative for obtaining actionable concentration data. In addition to analytical performance, the device offers significant practical advantages as a complete sensor system. Its intuitive readout facilitates rapid interpretation without specialized training. Crucially, the integrated Wi-Fi connectivity allows for the automated transmission of results (including concentration data, analysis time, and operator ID) directly to a central database or PC for systematic data management. This digital workflow fundamentally addresses the critical limitations of conventional rapid kits, which rely on subjective visual reading and cumbersome manual recording or photographic documentation. By ensuring objective data capture and secure transmission, our system significantly improves the reliability and evidentiary robustness required for legal and investigative purposes.

The future potential of this platform is considerable. The cartridge structure, which already integrates separate test and reference zones, is inherently suited to multiple assays. The clear and crucial next step is to incorporate a creatinine assay into a parallel channel. This enhancement addresses two critical aspects: sample validity testing (SVT) and con-centration normalization. In forensic drug testing, urine output varies significantly, and the concentration of drugs or their metabolites can be diluted or concentrated depending on the water content [26,27]. To address this variability, creatinine measurement is employed as a standard normalization method to correct for dilution effects [28]. Importantly, creatinine-normalized urinary metabolite concentrations have been shown to serve as a reliable proxy for blood drug concentrations [29].

Furthermore, sample validity testing (SVT) is crucial to prevent intentional tampering, such as sample dilution. The U.S. Government (SAMHSA) mandates validity testing and classifies specimens as follows: diluted urine (2–20 mg/dL creatinine) and substituted urine (≤2 mg/dL) [19]. In contrast, the World Health Organization (WHO) establishes a broader acceptable range of 30–300 mg/dL creatinine for valid urine specimens [30]. Implementation of an SVT assay aligned with these international forensic standards is es-essential for both preventing intentional sample manipulation and ensuring the analytical reliability of on-site drug testing results.

Finally, the successful validation utilizing THC-COOH demonstrates that the core integrated technology—combining sensitive microfluidic fluorescence analysis, normalized digital analysis, and secure automated data processing—functions as a robust and scalable sensor platform. This system is not limited to cannabis and can be readily extended to various analytes, ranging from other illicit drugs to clinical biomarkers, positioning it as a versatile and comprehensive solution for diverse point-of-care testing (POCT) applications.

## 5. Conclusions

The compact semi-quantitative analytical device developed in this study addresses the limitations of conventional rapid kits, achieving high accuracy and sensitivity in the field detection of THC-COOH. Importantly, the cutoff concentration for cannabis detection was set at 20 ng/mL, lower than the conventional 50 ng/mL threshold, allowing for the detection of lower THC-COOH levels. Evaluation with 100 authentic urine samples (50 positive and 50 negative by LC-MS/MS) demonstrated 100% sensitivity and 100% specificity. Furthermore, its semi-quantitative measurement capability was supported by a strong correlation (R^2^ = 0.9471) between its COI values and LC-MS/MS quantitative values. The developed device is portable, enables direct analysis of urine without complex pretreatment, and provides rapid, semi-quantitative results. Its user-friendly interface and integrated data management functions facilitate timely decision-making by field personnel while enhancing the evidentiary value of results for legal and regulatory purposes. These attributes make the device particularly suitable for applications such as roadside screening and workplace drug testing. Beyond cannabis, the underlying technology can be adapted for multi-drug screening, representing a versatile platform for public health and safety applications.

## Data Availability

The data presented in this study are available within the article and its Supplementary Material. Further inquiries can be directed to the corresponding author.

## Supplementary Materials

The following supporting information can be downloaded at https://www.mdpi.com/article/10.3390/s25237115/s1, Table S1: LC-MS/MS method validation—Between-run and within-run precision and accuracy for THC-COOH quantification using calibration standards and QC samples.; Table S2: LC-MS/MS method validation—extraction recovery for THC-COOH and internal standard (THC-COOH-d3); Table S3: LC-MS/MS method validation—matrix effect assessment for THC-COOH quantification.; Table S4: Comparison of device COI values and LC-MS/MS quantitative results for 50 authentic positive urine samples.

## Abbreviations

The following abbreviations are used in this manuscript:

BSAI	Bovine Serum Albumin
COI	Cutoff Index
CV	Coefficient of Variation
EDC	1-ethyl-3-(3-dimethylaminopropyl) carbodiimide
GC-MS/MS	Gas Chromatography-Tandem Mass Spectrometry
IS	Internal Standard
LC-MS/MS	Liquid Chromatography–Tandem Mass Spectrometry
LDT	Laboratory-Developed Test
LFA	Lateral Flow Assay
LOB	Limit of Blank
LOD	Limit of Detection
MES	2-(N-morpholino)-ethanesulfonic acid
MRM	Multiple Reaction Monitoring
NFS	National Forensic Service
NHS	N-hydroxysuccinimide
PBS	Phosphate-Buffered Saline
PMMA	Polymethyl methacrylate
POCT	Point-of-Care Testing
QC	Quality Control
RF	Radio Frequency
SCCM	Standard Cubic Centimeters per Minute
SVT	Specimen validity testing
THC	Tetrahydrocannabinol
THC-COOH	11-nor-9-carboxy-delta-9-tetrahydrocannabinol
WHO	World Health Organization
