# Supplementary material for: Semi-Quantitative On-Site Microfluidic Assay to Detect 11-Nor-9-carboxy-delta 9-Tetrahydrocannabinol (THC-COOH) in Urine
Source: Sensors (Basel). 2025 Nov 21;25(23):7115. doi: 10.3390/s25237115 (PMC12694267; doi:10.3390/s25237115)
Supplement: Supplementary file 1 [file sensors-25-07115-s001.zip › sensors-3982712-supplementary.pdf]

Table S1. LC-MS/MS method validation—Between-run and within-run precision and accuracy for THC-COOH quantification using calibration standards and QC samples.

Name	Calibration Curve Standard					Quality Control (within-run)				Quality Control (between-run)			
	1	2	3	4	5	LLOQ	LQC	MQC	HQC	LLOQ	LQC	MQC	HQC
Nominal concentration (ng/mL)	5	20	60	120	200	5	15	50	180	5	15	50	180
**THC-COOH(with Enzymatic treatment)**													
Mean (ng/mL)	4.990	20.10	60.83	120.4	196.0	5.461	15.95	52.29	179.5	5.206	15.88	52.26	180.0
Accuracy (%)	99.79	100.5	101.4	100.3	98.00	109.2	106.4	104.6	99.70	104.1	105.9	104.5	100.0
Precision (%)	1.808	8.333	2.367	1.445	4.401	4.337	3.991	3.184	0.6099	5.543	5.046	4.064	4.713
**THC-COOH(without Enzymatic treatment)**													
Mean (ng/mL)	5.044	19.02	62.02	123.4	195.7	5.383	16.22	53.81	183.7	5.326	15.79	52.62	181.8
Accuracy (%)	100.9	95.12	103.4	102.8	97.83	107.6	108.1	107.6	102.1	106.5	105.3	105.2	101.0
Precision (%)	0.4953	1.480	3.699	2.652	2.089	5.516	4.901	4.055	4.238	3.007	5.976	5.390	5.386

Table S2. LC-MS/MS method validation—Extraction recovery for THC-COOH and internal standard (THC-COOH-d3).

Name	Recovery	
	THC-COOH	THC-COOH-d_3 (IS)
**THC-COOH(with Enzymatic treatment)**		
Mean Recovery (%)	76.37	79.71
CV (%)	4.21	2.30
**THC-COOH(without Enzymatic treatment)**		
Mean Recovery (%)	91.40	91.14
CV (%)	2.45	5.84

Table S3. LC-MS/MS method validation—matrix effect assessment for THC-COOH quantification.

Name	Matrix effect	
	LQC	HQC
Nominal concentration (ng/mL)	15	180
**THC-COOH(with Enzymatic treatment)**		
Mean (ng/mL)	16.01	178.2
CV (%)	3.40	1.71
**THC-COOH(without Enzymatic treatment)**		
Mean Recovery (%)	15.79	196.5
CV (%)	2.87	2.13

Table S4. Comparison of device COI values and LC-MS/MS quantitative results for 50 authentic positive urine samples.

Sample No.	Absol device	LC-MS/MS	
	COI value	THC-COOH (ng/mL) with Enzymatic treatment	THC-COOH (ng/mL) without Enzymatic treatment
Sample-01	766.6	10,950	2,458
Sample-02	20.32	172.4	36.23
Sample-03	83.07	442.8	349.6
Sample-04	4.097	36.06	7.246
Sample-05	42.33	80.84	52.17
Sample-06	1.229	21.50	7.957
Sample-07	111.8	1,916	740.7
Sample-08	1.541	15.09	NQ(2.173)
Sample-09	2.904	29.17	5.257
Sample-10	7.144	85.42	19.51
Sample-11	38.90	435.4	187.8
Sample-12	39.51	311.9	80.81
Sample-13	1.722	33.56	NQ(3.406)
Sample-14	11.85	68.76	34.17
Sample-15	1.316	7.557	NQ(1.073)
Sample-16	24.20	439.1	156.9
Sample-17	5.278	68.91	26.93
Sample-18	8.702	97.27	36.07
Sample-19	1.470	19.20	6.729
Sample-20	5.770	47.54	NQ(3.848)
Sample-21	18.80	220.9	67.55
Sample-22	61.91	555.0	59.68
Sample-23	22.42	172.8	30.70
Sample-24	1.263	17.87	NQ(0.6779)
Sample-25	1.110	10.54	NQ(1.164)
Sample-26	88.54	1,303	562.6
Sample-27	4.426	51.99	6.573
Sample-28	11.54	128.5	16.87
Sample-29	25.66	280.2	6.715
Sample-30	69.26	593.8	79.92
Sample-31	21.76	296.6	106.2
Sample-32	5.283	50.43	12.07
Sample-33	3.655	27.11	NQ(4.535)
Sample-34	1.529	18.55	NQ(2.822)
Sample-35	196.9	2,712	1,193
Sample-36	5.558	77.76	10.05
Sample-37	4.561	47.48	23.27
Sample-38	31.60	875.6	175.8
Sample-39	1.580	22.13	6.650
Sample-40	24.72	182.7	35.71
Sample-41	2.776	24.66	8.401
Sample-42	6.226	51.79	16.24
Sample-43	141.2	578.0	89.45
Sample-44	125.4	2,000	102.9
Sample-45	13.38	118.4	36.11
Sample-46	175.9	2,557	1,837
Sample-47	6.412	33.49	9.669
Sample-48	174.8	1,584	942.0
Sample-49	9.934	119.8	29.61
Sample-50	246.3	1,565	153.0

*** NQ: Not Quantifiable**
